# Detection of nitrite based on fluorescent carbon dots by the hydrothermal method with folic acid

**DOI:** 10.1098/rsos.172149

**Published:** 2018-05-02

**Authors:** Haitao Lin, Liyun Ding, Bingyu Zhang, Jun Huang

**Affiliations:** National Engineering Laboratory for Fiber Optic Sensing Technology, Wuhan University of Technology, Wuhan 430070, People's Republic of China

**Keywords:** nitrite detection, carbon dots, fluorescence quenching

## Abstract

A fluorescent carbon dots probe for the detection of aqueous nitrite was fabricated by a one-pot hydrothermal method, and the transmission electron microscope, X-ray diffractometer, UV–Vis absorption spectrometer and fluorescence spectrophotometer were used to study the property of carbon dots. The fluorescent property of carbon dots influenced by the concentration of aqueous nitrite was studied. The interaction between the electron-donating functional groups and the electron-accepting nitrous acid could account for the quenching effect on carbon dots by adding aqueous nitrite. The products of the hydrolysis of aqueous nitrite performed a stronger quenching effect at lower pH. The relationship between the relative fluorescence intensity of carbon dots and the concentration of nitrite was described by the Stern–Volmer equation (*I*_0_/*I *−* *1 = 0.046[*Q*]) with a fine linearity (*R*^2^ = 0.99). The carbon dots-based probe provides a convenient method for the detection of nitrite concentration.

## 1. Introduction

Nitrite as an important oxynitride plays a significant role in the nitrogen cycle of the natural environment. Meanwhile, in the fields of agriculture and the food industry, it is regularly used as fertilizer and as a preservative, respectively. However, the usage of nitrite is constrained in physiological systems because the excessive ingestion of nitrite can lead to hazardous effects for human health, especially for children, the elderly and pregnant women [1–5]. For example, haemoglobin oxidation in blood, the formation of carcinogenic nitrosamines in the digestive system [6,7] and many diseases such as oesophageal cancer and blue baby syndrome [8–10] are all caused by an excess ingestion of nitrite. The World Health Organization specifies that the maximum concentration of nitrite should not exceed 3 mg l^−1^ in drinking water [11]. Therefore, it is important to detect nitrite in the living environment to improve the quality of human health. In recent years, many methods of aqueous nitrite determination have entered daily life, including spectrophotometry [12,13], electrochemistry [14,15], chemiluminescence [16–18] and fluorometric methods. Afkhami *et al.* [12] reported the spectrophotometric determination of nitrite based on the colour reaction of nitrite with *p*-nitroaniline. The electrochemical nitrite sensor based on graphene quantum dots decorated N-doped carbon nanofibres composite was found by Li *et al.* [19]. Abdolmohammad-Zadeh & Rahimpour [20] used ag@agcl@graphene oxide@Fe_3_O_4_ nanocomposite for chemiluminescent detection of nitrite. However, their work still had some disadvantages, such as complicated and expensive instruments, toxic raw materials and a complicated synthetic process. Compared with these methods, fluorometric methods have many advantages including simple operation, speediness, low cost, portability, high sensitivity, good selectivity and high time efficiency, as shown in table 1. Liu *et al.* [21] used fluorometric methods based on graphene quantum dots co-doped for nitrite sensing.

**Table 1.** Methods in the field of nitrite detection.

method	feature
spectrophotometry [12,13]	strictly reaction conditions; easily disturbed reaction process
electrochemistry [14,15]	expensive electrode; unsatisfactory trace detection
chemiluminescence [16–18]	not suitable for field-based testing
fluorometry [23]	selectivity, portability and time efficiency; non-toxicity for environment; easy to prepare; low cost; high sensitivity

For fluorescence detection, it is well known that the commonly used organic dyes often suffer from light bleaching. A solid substitute is fluorescent semiconductor quantum dots, such as CdTe, CdS and PbS, which are a kind of ‘zero-dimensional’ nano-material with a quantum confinement effect. However, the fluorescent semiconductor quantum dots are often synthesized with heavy metal ions, which could substantially diminish their biocompatibility. Carbon dots, as a relatively new member of the nano-material family [22], inherit some of the good properties of semiconductor quantum dots such as photostability, and are also capable of maintaining non-toxicity, with a green synthesis from reagents to products. Therefore, they have potential in bioimaging, biosensing and nanomedicine [23]. It has been reported that carbon dots were used as reductant and reinforcement for hydrogen peroxide [24] and nitrite detection [25].

In this paper, an aqueous nitrite sensor was fabricated based on the fluorescent carbon dots synthesized by the hydrothermal method. The character of carbon dots was studied by transmission electron microscope (TEM), X-ray diffractometer (XRD), UV–Vis absorption spectrometer and fluorescence spectrophotometer. The fluorescence of carbon dots could be quenched by aqueous nitrite because of the electron transfer between the electron-donating functional groups on the surface of the carbon dots and the electron-accepting nitrous acid. The results showed that aqueous nitrite performed a fierce quenching effect on the fluorescence of the carbon dots at lower pH. The effects of synthesis temperature, pH and the interference effect of other ions for carbon dots were studied in detail.

## 2. Material and methods

### 2.1. Materials

Folic acid (AR), NaOH (AR), H_2_O_2_ (AR), NaH_2_PO_4_ (AR), NH_4_F (AR), KCl (AR), NaBr (AR), K_2_SO_4_ (AR) and NaNO_3_ (AR) were provided by Sinopharm Chemical Reagent Co. Deionized water was generated by the Hitech-K flow water purification system.

### 2.2. Synthesis of carbon dots

The method of synthesis of carbon dots was the hydrothermal process [26]. Folic acid (1 g) and 6 mol l^−1^ NaOH (0.9 ml) were dissolved in deionized water (20 ml) under agitation. The obtained solution was then heated at 170, 180, 190 and 200°C for 5 h in a 50 ml hydrothermal reactor. When the reaction was finished, the reactors were allowed to cool to ambient temperature naturally. The obtained brown solution was centrifuged at 9000 rpm for 8–15 min to remove impurities and filtered using a pinhole filter. The prepared carbon dots were kept at approximately 4°C in a refrigerator.

### 2.3. Characterization

The fluorescence spectrophotometer (F-4500, HITACHI, Japan) and the UV-2450 spectrometer (Shimadzu, Japan) were selected to record the fluorescence spectra and the absorption spectra of carbon dots, respectively. The JEM 2100F STEM (JEOL, Japan) and the XRD (D8 Advance, Bruker, Germany) were used to record TEM image and XRD patterns of the carbon dots, respectively.

## 3. Results and discussion

### 3.1. The characterization of carbon dots

**Figure 1.**
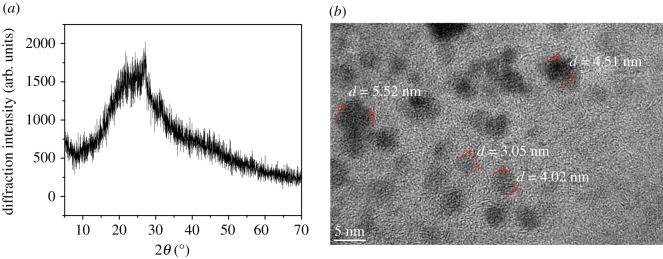
(*a*) The XRD pattern of carbon dots. (*b*) The high-resolution transmission electron microscopy images of carbon dots.

**Figure 2.**
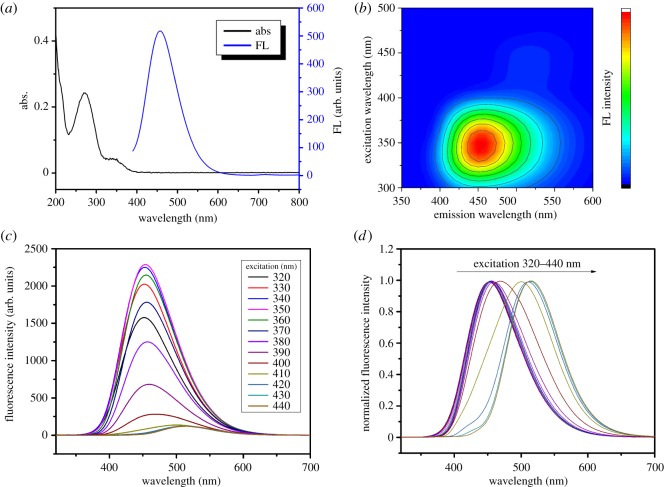
(*a*) The absorption spectrum of carbon dots. (*b*) The three-dimensional scanning fluorescence spectra of carbon dots. (*c*) The fluorescence spectra of carbon dots excited at various wavelengths. (*d*) The normalized fluorescence spectra of carbon dots.

The XRD pattern of carbon dots is shown in figure 1*a*. The peak at 25° corresponds to the crystal lattice distance of (002), which is a key feature of materials based on carbon [27]. TEM of carbon dots in figure 1*b* shows that the size of the carbon dots was in the range of 3–6 nm and the crystal structure was observed unclearly, which demonstrated that the structure of the carbon dots was dominated by the amorphous form. The UV–Vis absorption and fluorescence spectra of the carbon dots were recorded in figure 2*a*. The electronic transition of C–C and C=O bonds in the excited carbon dots could account for the peaks at 261 nm and 343 nm in the absorption spectrum, respectively. It can be seen that the optimum excitation wavelength of the carbon dots was 355 nm.

Figure 2*b* shows the fluorescence emission intensity of the carbon dots, and demonstrates that the strongest emission wavelength of the carbon dots was located in the range of 440–475 nm at the excitation wavelength in the range of 330–365 nm. From figure 2*c*, it can be seen that the different wavelength excitation of the carbon dots could account for the varying fluorescence intensity. The spectra of the carbon dots were normalized to show that excitation at different wavelength also resulted in fluorescence wavelength shift as shown in figure 2*d*. The wavelength of emission shifted from 450 to 505 nm when the excitation shifted from 320 to 440 nm, which might be assigned to the various sizes for carbon cores and different surface groups. It has been widely reported that multiple fluorophores exist on the surface of carbon dots, contributing to a wide range of emission wavelength [28]. However, under certain monocolour excitation, most fluorophores would be eclipsed by the dominant ones. This dominating behaviour largely depends on the absorbance and quantum yield of the fluorophores. So as the excitation wavelength varies from short to long, the fluorophores with high absorbance around the current wavelength take the stage successively. As a result, the emission wavelength shows a dependence on the excitation. Many applications based on carbon dots can benefit from the property that they have a wide range of excitation and emission wavelengths.

### 3.2. The fluorescent properties of the carbon dots

**Figure 3.**
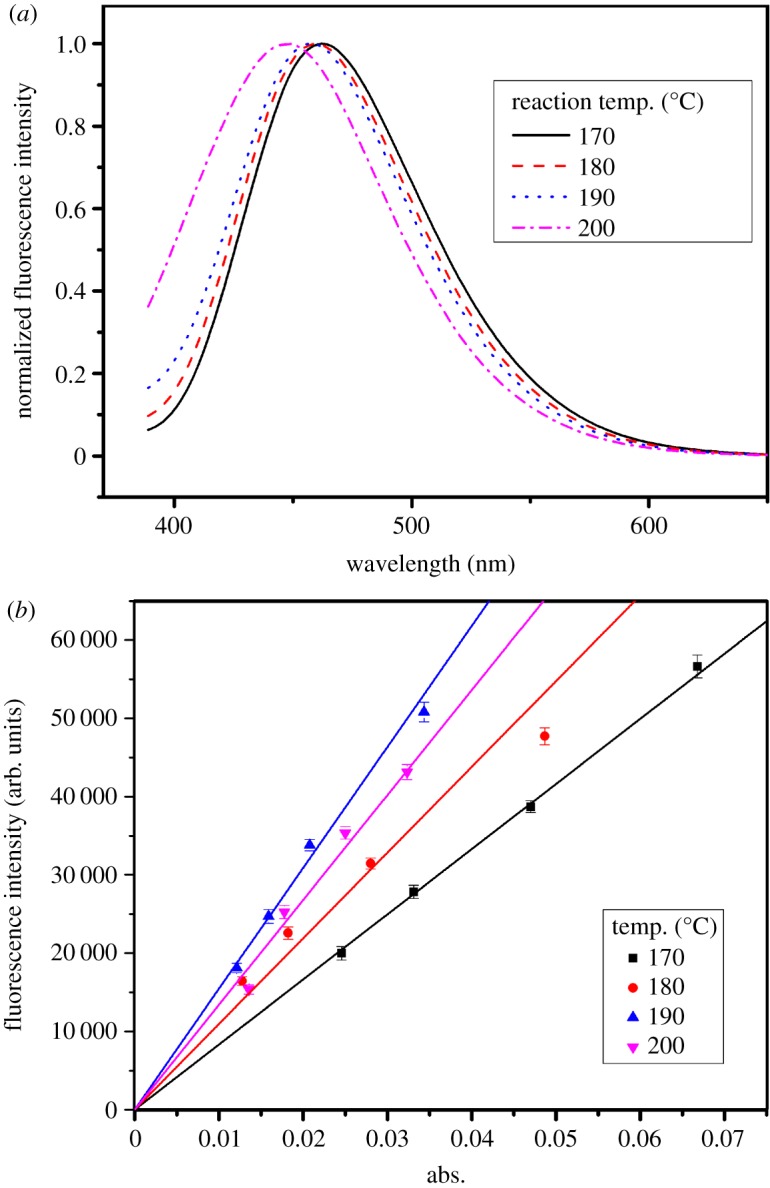
(*a*) Normalized fluorescence intensity and (*b*) quantum yield of carbon dots prepared under different temperatures.

The normalized fluorescent spectra of the carbon dots shown in figure 3*a* illustrate that the emission wavelength red shifted from 447 to 462 nm when the temperature of synthesis decreased from 200°C to 170°C, indicating that the degree of carbonization of carbon decreases with the decrease of temperature, leading to the proportional increase of surface sites. The shift of fluorescence peak is due to the fluorescent peak of the carbon dots formed by the superposition of multiple fluorophores and weaker transition energy of the surface groups than that of the carbon skeleton. Because of the blue fluorescence of carbon dots, quinine sulfate in 0.1 mol l^−1^ H_2_SO_4_ aqueous solution was selected as a standard fluorescent agent. Absolute values were calculated according to the following equation:

$$Q_{x}=Q_{\mathrm{st}}\left(\frac{M_{x}}{M_{\mathrm{st}}}\right){\left(\frac{\gamma_{x}}{\gamma_{\mathrm{st}}}\right)}^{2}$$

where *Q* is the quantum yield, *M* is the gradient from the linear regression and *γ* is the refractive index of the solution. The subscripts ‘st‘ and ‘*x*‘ refer to standard fluorescent agent and sample, respectively. The ratio of the refractive index of solvent was 1. To ensure a linear relationship between the absorbance of solution and the molar concentration of solution, the absorbance of solvent was kept under 0.1 at the 350 nm excitation. The relationship between the fluorescence integral intensity and absorbance (355 nm) shown in figure 3*b* indicates that the straight slope expressed the fluorescence quantum yield of carbon dots. As can be seen, changes in the temperature of preparation will lead to changes in the fluorescence yield. In the range from 170°C to 200°C, carbon dots prepared at 190°C had the highest quantum yield. The heating temperature directly affects the degree of carbonization reaction, and the heating temperatures are important factors influencing the final product characteristics. Low temperature leads to insufficiency of carbon centre crystal growth. Excessive high temperature carbonization of raw materials will reduce organic groups on the surface of carbon dots, which will lower their fluorescence [29]. Therefore, the optimum temperature of 190°C was used in our experiments.

### 3.3. The fluorescence of carbon dots quenched by aqueous nitrite

**Figure 4.**
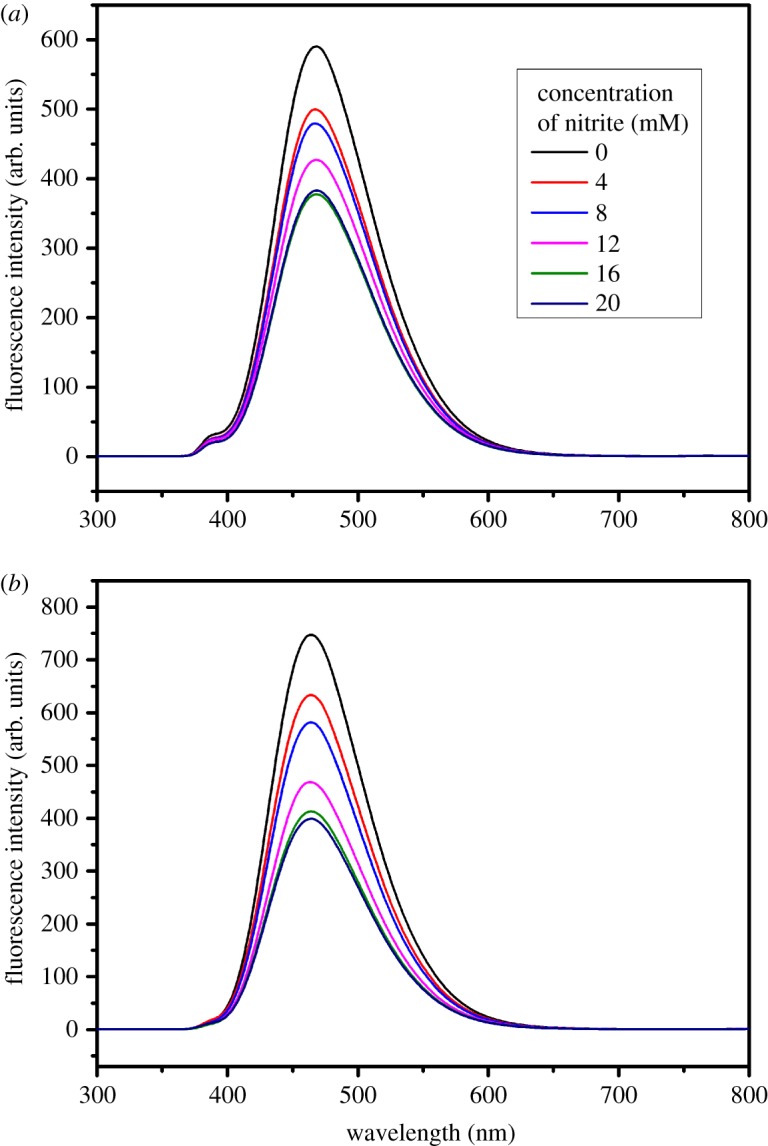
Fluorescence spectra of carbon dots varied concentrations of aqueous nitrite under pH 7 (*a*) and pH 5 (*b*).

The fluorescence quenching of carbon dots affected by the aqueous nitrite under pH 7 and 5 is shown in figure 4*a*,*b*, respectively. When the reaction between carbon dots and aqueous nitrite occurs, aqueous nitrite can be regarded as an electron acceptor, so some of the electrons of the carbon dots received the photon energy and were transformed to an excited state and then were transferred to the acceptor. Therefore, the fluorescence intensity of the carbon dots was reduced on a macroscopic scale. This property of carbon dots was advantageous for nitrite detection in the water sample.

**Figure 5.**
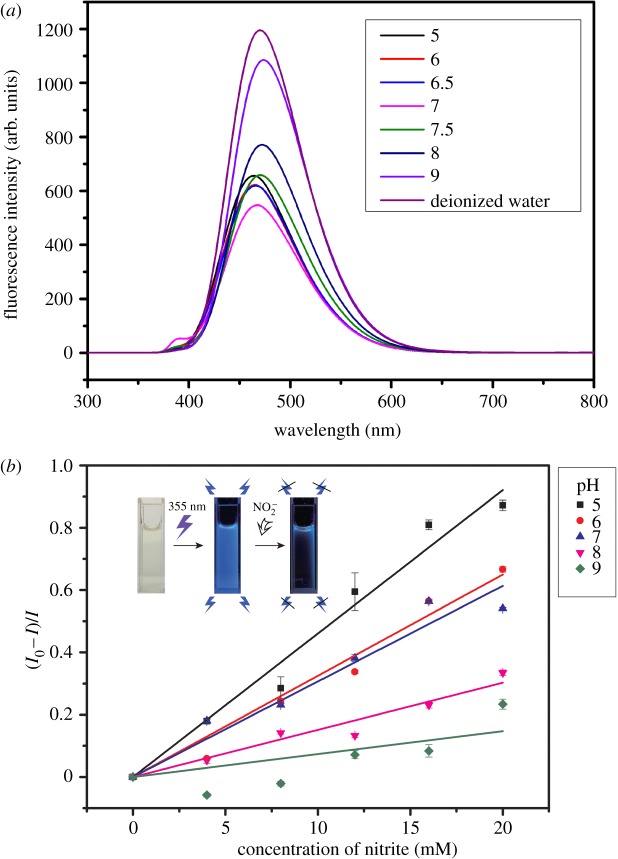
(*a*) Fluorescence spectroscopy of carbon dots under different pH values and (*b*) the Stern–Volmer plot for the quenching of the carbon dots caused by aqueous nitrite.

The fluorescence intensities and peaks of carbon dots varied with the change in pH as shown in figure 5*a*. The reason might be ascribed to the influence of the concentration of hydrogen ion in aqueous solution upon the fluorescent groups on the surface of carbon dots. There was a certain linear relationship between relative fluorescence intensity of the carbon dots and the concentration of aqueous nitrite under different pH values as shown in figure 5*b*. The carbon dots solution (see images in the upper left corner of figure 5*b*) changes from colourless to bright blue after excitation by UV light at 355 nm. With increasing concentration of the aqueous nitrite, the fluorescence intensity of the carbon dots decreased accordingly. The influence of the same concentration of aqueous nitrite on the carbon dots was different under various pH values. The fluorescence intensity of the carbon dots was more sensitive to nitrite with the decrease of pH. The following Stern–Volmer equation was used to analyse the fluorescence quenching of aqueous nitrite:

$$I_{0}/I-1=K_{\mathrm{sv}}[C],$$

where *I_0_* and *I* are the fluorescence intensity of carbon dots without and with the aqueous nitrite, respectively, *K*_sv_ is the quenching constant which is the slope of the linearity and [*C*] is the concentration of the aqueous nitrite. The fine linearity *I*_0_/*I − *1 = 0.046[NaNO_2_] (*R*^2^ = 0.99) was achieved under pH 5 in figure 5*b*. The sensitivity of fluorescent carbon dots for detection of aqueous nitrite can be described by *K*_sv_. A higher value of *K*_sv_ indicates that the more obvious quenching effect can be observed at a slighter change in the concentration of the aqueous nitrite. Because nitrous acid is a weak acid, hydrolysis reaction of nitrite ion exists in water. The hydrolysis reaction of nitrite ion was reinforced under the acidic condition. Therefore, the content of nitrous acid in aqueous solution increased, which enhanced the effect of fluorescence quenching due to the higher activity of nitrous acid than nitrite ion.

**Figure 6.**
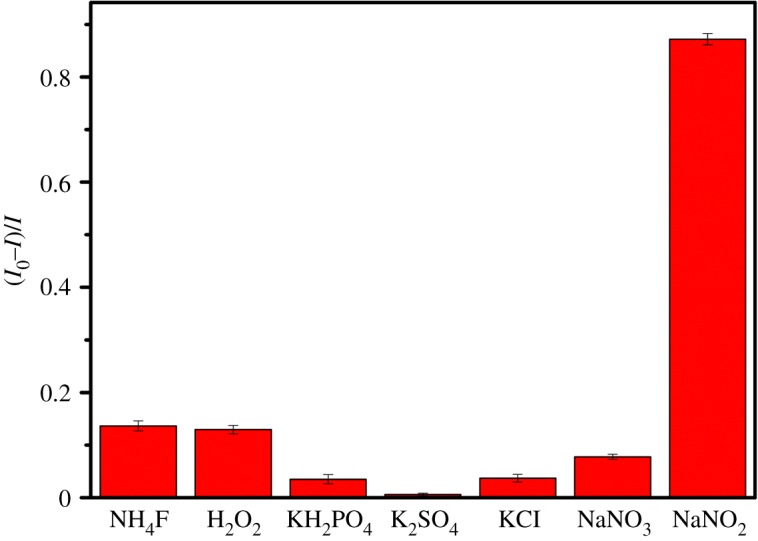
The changes in fluorescence intensities of the carbon dots after the addition of different chemicals (20 mM).

Different chemicals were added into the solution of carbon dots to study the interference effect of other ions on fluorescent carbon dots. Figure 6 shows the results of the fluorescence intensity of carbon dots after the addition of varies chemicals (20 mM). Compared with aqueous nitrite, much less change in the fluorescence of carbon dots was brought about by other ions including NH4^+^, F^−^, H_2_O_2_, K^+^, ${\text{H}}_{2}\text{P}{{\text{O}}_{4}}^{-}$, $\text{S}{{\text{O}}_{4}}^{2-}$, Cl^−^, Na^+^ and NO^3–^, which demonstrated that the detection based on carbon dots had obvious specificity.

## 4. Conclusion

In conclusion, the hydrothermal approach was used to synthesize carbon dots with good photostability. The fluorescence intensity of carbon dots was testified to decline with increased concentration of aqueous nitrite under various pH values. With the decrease of pH in the range from 9 to 5, the fluorescence quenching effect of aqueous nitrite became stronger. The fine linearity *I*_0_/*I* − 1 = 0.046[NaNO_2_] (*R*^2^ *=* 0.99) was achieved, which demonstrated that the concentration of aqueous nitrite could be detected by this linearity given certain changes of fluorescence intensity of carbon dots. The carbon dots demonstrated a good selective recognition ability and could be a candidate for a fluorescence probe for nitrite detection. Furthermore, their biocompatibility, non-toxicity, rapid response and stable photoluminescence were advantageous to their applications in several fields.

## Data accessibility

All original data have been deposited at the Dryad Digital Repository: https://doi.org/10.5061/dryad.8vd0gf1 [30].
